# BMAL1 Modulates Epidermal Healing in a Process Involving the Antioxidative Defense Mechanism

**DOI:** 10.3390/ijms21030901

**Published:** 2020-01-30

**Authors:** Ericka J. D. Silveira, Carlos H. V. Nascimento Filho, Veronica Q. Yujra, Liana P. Webber, Rogerio M. Castilho, Cristiane H. Squarize

**Affiliations:** 1 Laboratory of Epithelial Biology, Department of Periodontics and Oral Medicine, University of Michigan School of Dentistry, Ann Arbor, MI 48109, USA; ericka_janine@yahoo.com.br (E.J.D.S.); cviesido@umich.edu (C.H.V.N.F.); veroniqy96@gmail.com (V.Q.Y.); lpretowe@umich.edu (L.P.W.); rcastilh@umich.edu (R.M.C.); 2 Odontology Sciences Postgraduate Program, Dentistry Department, Federal University of Rio Grande do Norte, Natal 59056, RN, Brazil; 3 The Michigan Medicine Rogel Cancer Center, University of Michigan, Ann Arbor, MI 48109, USA

**Keywords:** ROMO1, clock genes, skin, SOD1, BMAL1

## Abstract

The circadian rhythm regulates the physiology and behavior of living organisms in a time-dependent manner. Clock genes have distinct roles including the control over gene expression mediated by the transcriptional activators CLOCK and BMAL1, and the suppression of gene expression mediated by the transcriptional repressors PER1/2 and CRY1/2. The balance between gene expression and repression is key to the maintenance of tissue homeostasis that is disrupted in the event of an injury. In the skin, a compromised epithelial barrier triggers a cascade of events that culminate in the mobilization of epithelial cells and stem cells. Recruited epithelial cells migrate towards the wound and reestablish the protective epithelial layer of the skin. Although we have recently demonstrated the involvement of BMAL and the PI3K signaling in wound healing, the role of the circadian clock genes in tissue repair remains poorly understood. Here, we sought to understand the role of BMAL1 on skin healing in response to injury. We found that genetic depletion of BMAL1 resulted in delayed healing of the skin as compared to wild-type control mice. Furthermore, we found that loss of Bmal1 was associated with the accumulation of Reactive Oxygen Species Modulator 1 (ROMO1), a protein responsible for inducing the production of intracellular reactive oxygen species (ROS). The slow healing was associated with ROS and superoxide dismutase (SOD) production, and pharmacological inhibition of the oxidative stress signaling (ROS/SOD) led to cellular proliferation, upregulation of Sirtuin 1 (SIRT1), and rescued the skin healing phenotype of *Bmal1*^−/−^ mice. Overall, our study points to BMAL1 as a key player in tissue regeneration and as a critical regulator of ROMO1 and oxidative stress in the skin.

## 1. Introduction

The circadian clock is an internal time-keeping system conserved across species and involved in the regulation of physiological processes, including metabolism [1]. Clock genes are also crucial components of tissue development, physiological turnover, and cytokine production, among other biological processes [2,3,4,5,6,7]. Disruptions to the circadian clock are associated with increased chances of developing diabetes, cardiovascular disease, neural and retinal degeneration, muscle diseases, osteoarthritis, and cancer [8,9,10,11,12,13,14,15]. Clock genes are also involved with the repair of DNA and tumor response to radiotherapy and chemotherapy [16,17].

At the molecular level, the circadian clock is regulated by a complex of transcriptional activators (CLOCK, BMAL1) and repressors (PER1/2 and CRY1/2). These effectors can control cellular functions, such as cellular adaptive response [1,18]. The clock gene BMAL1 (also known as Brain and Muscle ARNT-Like 1, Aryl hydrocarbon receptor nuclear translocator-like protein 1, or ARNTL) is a transcriptional factor with known association with premature aging, decreased fertility, and shorter life span [19,20]. BMAL1 is found ubiquitously across species. Thus, it is expressed in normal and malignant epithelial cells [21,22]. Although there are pieces of evidence on BMAL1 participation in skin homeostasis, its role in skin repair remains poorly understood.

Here, we investigated the effects of BMAL1 during skin response to injury using a well-established skin injury model. Our results show that BMAL1 plays an important role in epithelial proliferation, migration, and wound closure. Depletion of BMAL1 negatively impacts epidermal healing and triggers the buildup of Reactive Oxygen Species Modulator 1 (ROMO1) and intracellular accumulation of reactive oxygen species (ROS). Notably, topical application of *N*-Acetyl-l-cysteine (NAC) reversed the deleterious effects of ROS accumulation and rescued the BMAL1 slow-healing phenotype.

## 2. Results

### 2.1. Depletion of Bmal1 Delays Epidermal Healing

**Figure 1:**
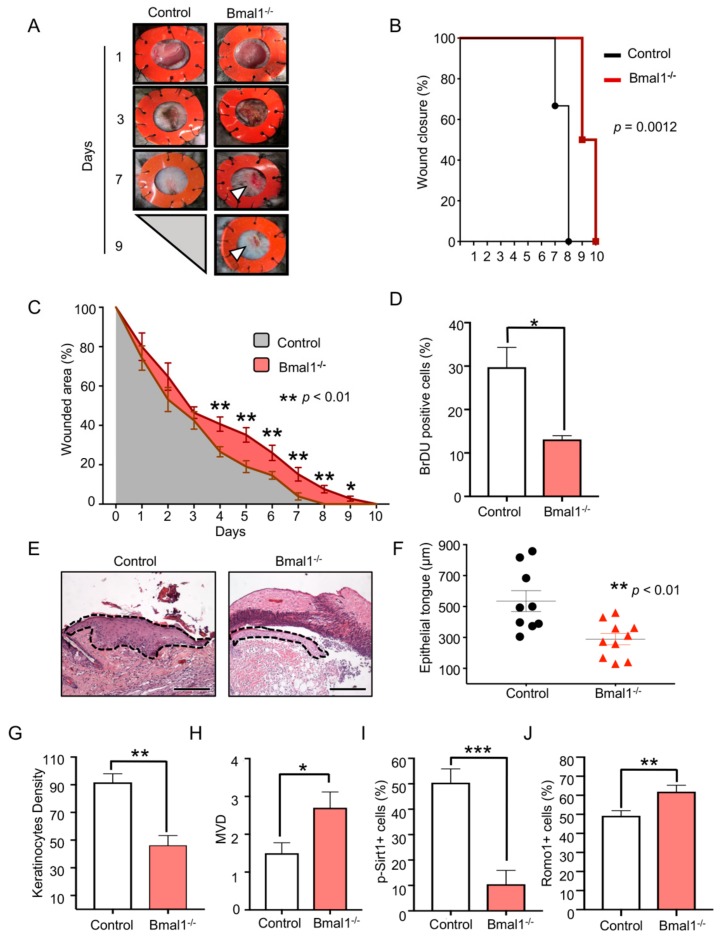
*Bmal1* deletion affects Reactive Oxygen Species Modulator 1 (ROMO1) and phosphorylation of SIRT1 (p-SIRT1) activation and delays epithelial wound healing. (**A**) Representative photographs of wounds from *Bmal1*^−/−^ and wild-type mice fitted with silicone splints at days 1, 3, 7, and 9 post-injury. (**B**) Graphic depicts the percentage of mice exhibiting wound closure identified among the control group (black line) and *Bmal1*^−/−^ (red line) at indicated days. Note significant longer healing time for *Bmal1*^−/−^ mice compared to wild-type mice (** *p* = 0.0012). (**C**) Open wound area of *Bmal1*^−/−^ and wild-type mice during daily follow-up (*n* = 10/time point and group, percentage mean ± SEM (error bar), ** *p* < 0.01). (**D**) Quantification of bromodeoxyuridine (BrdU) positive cells present in the *Bmal1*^−/−^ and wild-type mice (percentage mean ± SEM, * *p* < 0.05). The tissues were collected at day 5 post-injury. (**E**) Representative hematoxylin and eosin (H&E) stained skin wounds depicting the re-epithelialization of wounds on *Bmal1*^−/−^ and wild-type mice. The black, dashed line delineates the migrating epithelial tongue over the wounded area (scale bar, 200 µm). (**F**) Dot plot graphic of the epithelial tongue length from *Bmal1* and wild-type mice (mean ± SEM, ** *p* < 0.01). (**G**) Quantification of the keratinocytes presented in the epithelial tongues (mean ± S.E.M, *n* = 5 mice/group, ** *p* < 0.01). (**H**) Quantification of the microvessel density (MVD) of wounds from *Bmal1*^−/−^ mice compared to wild-type mice (mean ± SEM, * *p* < 0.05). (**I**) The graphic shows the percentage of p-SIRT1 positive cells on *Bmal1*^−/−^ mice and wild-type (percentage mean ± SEM, *** *p* < 0.001). (**J**) Graphic shows the percentage of ROMO1 positive cells in skin epithelial cells (percentage mean ± SEM, ** *p* < 0.01).

To determine the contribution of the BMAL1 to the regenerative process of the skin, we first analyzed the closure of skin wounds on the *Bmal1^−/−^*mice. To avoid skin contraction, splints (silicone rings) were placed around circular wounds (Figure 1A). The healing process of skin wounds was monitored daily. Notably, we observed that *Bmal1^−/−^*mice healed slower than the wild-type mice (Figure 1A,B, ** *p* = 0.0012). The significant changes became clinically noticeable starting at day 4 post-injury (Figure 1C, ** *p* < 0.01), and the complete wound closure of *Bmal1^−/−^* mice was two days behind the wild-type mice (Figure 1B,C). Next, we asked whether changes in proliferation might be affecting the healing of these mice. Using short pulse bromodeoxyuridine (BrdU) incorporation assay, we observed that *Bmal1^−/−^*mice had a significant reduction in the number of proliferating epithelial cells (Figure 1D, * *p* < 0.05). Additionally, we found that *Bmal1^−/−^*mice presented with a reduced epithelial coverage of the wounds (Figure 1E,F, ** *p* < 0.01) and a reduced number of epithelial cells located at the epithelial tongue compared to wild-type mice (Figure 1G, ** *p* < 0.01). Combined, the histological measurements of the epithelial tongue length and thickness ascertained that the re-epithelialization process of *Bmal1^−/−^*was delayed (Figure 1F,G). We also found that the neovascularization of the wound bed was affected by *Bmal1^−/−^* (Figure 1H, * *p* < 0.05). In search of altered pathways involved in the delayed healing process, we investigated whether the ablation of *Bmal1* would affect the Sirtuin 1 (SIRT1), a class III histone deacetylase enzyme, also known as NAD-Dependent Protein Deacetylase Sirtuin-1. SIRT1 is recognized for regulating cell proliferation via modulation of cyclin-dependent kinases (CDK) proteins. Notably, we found that phosphorylation of SIRT1 (p-SIRT1) was mostly absent in the nucleus of epithelial cells from *Bmal1^−/−^*mice (Figure 1I, *** *p* < 0.001). The process of wound healing depends on several factors, including the accumulation of small concentrations of ROS (physiological levels) [23]. ROS is also associated with the recruitment of lymphocytes and with the formation of blood vessels [24]. Here, we show that depletion of Bmal1 from epithelial cells results in the accumulation of ROMO1 (i.e., Reactive Oxygen Species Modulator 1), an inducer of ROS production (Figure 1J, ** *p* < 0.01).

### 2.2. Bmal1^−/−^ Mice Present High Levels of ROS and SOD upon Injury

**Figure 2:**
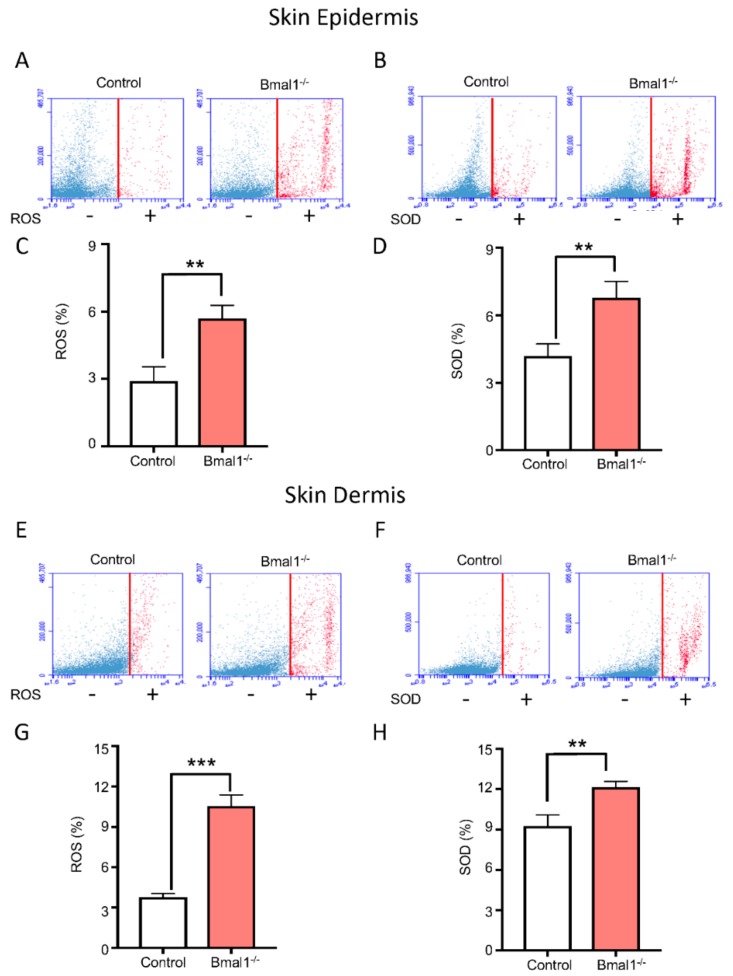
*Bmal1* depletion triggers the accumulation of reactive oxygen species (ROS) and superoxide dismutase (SOD) in skin cells. (**A**,**B**) Schematic representation of flow cytometry assay showing quantification of ROS and SOD on epithelial cells derived from wild-type and *Bmal1*^−/−^ mice as indicated. Note the positive cell population in red. (**C**,**D**) Graphics depict statistically significant accumulation of ROS and SOD in epithelial cells for ROS and SOD from *Bmal1*^−/−^ and wild-type mice (percentage mean ± SEM, ** *p* < 0.01). (**E**,**F**) Schematic representation of flow cytometry assay for ROS and SOD levels on connective tissue-derived cells from wild-type and *Bmal1*^−/−^ mice. (**G**,**H**) Graphics show the accumulation of ROS and SOD in cells derived from the connective tissue (percentage mean ± SEM, ** *p* < 0.01, *** *p* < 0.001).

Following our findings on the deregulation of SIRT1 and ROMO1 during skin wound healing process of *Bmal1^−/−^* mice, we hypothesized that *Bmal1* may be involved in the skin production of ROS and superoxide dismutase (SOD). To test this hypothesis, we analyzed whether skin from *Bmal1^−/−^*mice displayed any changes in ROS production upon injury. As shown in Figure 2, ROS and SOD levels were higher in the animals with *Bmal1* deletion compared to wild-type mice (Figure 2A–D, ** *p* < 0.01). Similar to the epidermis, the dermis from *Bmal1^−/−^*mice also presented elevated levels of ROS and SOD (Figure 2E–H, ** *p* < 0.01, *** *p* < 0.001). These results indicated that loss of function of the circadian gene *Bmal1* resulted in a significant increase of ROMO1 and elevated production of ROS and SOD in the skin.

### 2.3. Topical Delivery of NAC Halts ROS and SOD Accumulation Driven by BMAL1 Depletion

**Figure 3:**
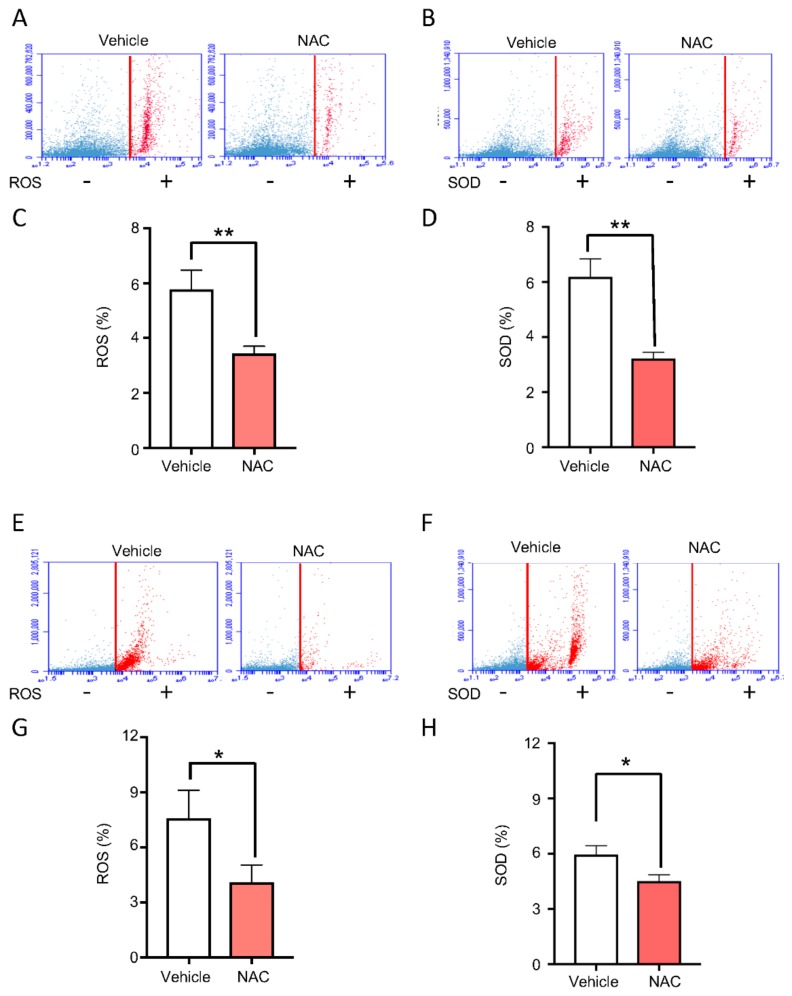
*N*-Acetyl-l-cysteine (NAC) reduces ROS and SOD levels from *Bmal1*^−/−^ skin cells. (**A**,**B**) Representative flow cytometry data for ROS and SOD fluorescent markers on epithelial cells of *Bmal1*^−/−^ mice treated with NAC or vehicle (positive cell population in red). (**C**,**D**) Graphic depicts epithelial cells from *Bmal1*^−/−^ mice treated with 3% NAC topical gel. Note reduced levels of ROS and SOD on NAC-treated skin when compared to vehicle treatment (percentage mean ± SEM, ** *p* < 0.01). (**E**,**F**) Representative flow cytometry analysis of ROS and SOD levels present on the connective tissue-derived cells from *Bmal1*^−/−^ mice receiving NAC and vehicle treatments. (**G**,**H**) Stromal-derived cells from *Bmal1*^−/−^ mice treated with NAC showing reduced levels of ROS and SOD when compared to vehicle (percentage mean ± SEM, * *p* < 0.05).

Once we had determined the importance of BMAL1 in the process of wound healing and accumulation of ROS, we sought to determine whether excessive ROS could be directly associated with the *Bmal1^−/−^* phenotype (i.e., delayed wound healing). Thus, we treated *Bmal1^−/−^* mice with topical applications of ROS inhibitor (3% NAC gel) shortly after skin wounding. Here, we first analyzed whether topical delivery of NAC would affect ROS levels in the skin. Our data showed a significant reduction of ROS and SOD levels on the epidermal (Figure 3A–D, ** *p* < 0.01) and, to a lower extent, the dermal compartments (Figure 3E–H, * *p* < 0.05) after topical administration of NAC.

### 2.4. Administration of NAC Rescues Bmal1^−/−^ Healing Phenotype

**Figure 4:**
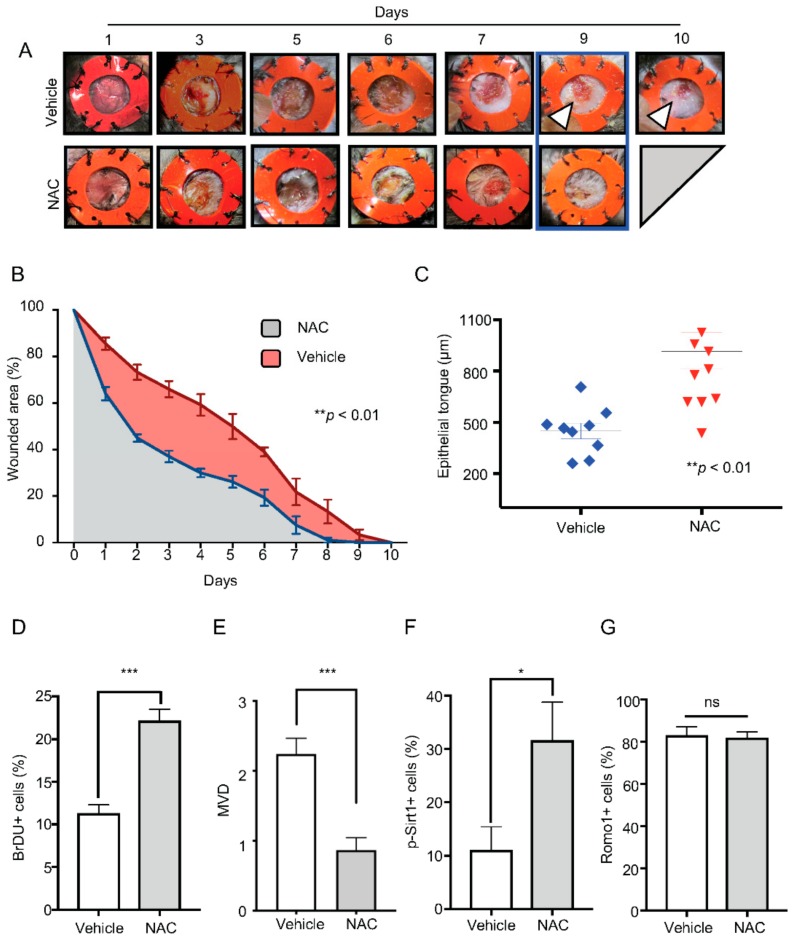
Administration of NAC rescues the *Bmal1*^−/−^ phenotype. (**A**) Representative photographs of *Bmal1*^−/−^ showing gradual wound closure on mice treated with daily topical applications of NAC or vehicle. (**B**) Graphic shows the percentage of open wound area on *Bmal1*^−/−^ mice distributed by days and treatment (NAC: gray, vehicle: red) (percentage mean ± SEM, *n* = 5/group, ** *p* < 0.01). (**C**) Quantification of epithelial tongue length from mice receiving NAC or vehicle. Note accelerated healing time for *Bmal1*^−/−^ mice receiving NAC (mean ± SEM, *n* = 9/treatment, ** *p* < 0.01). (**D**) Quantification of BrdU positive cells from mice receiving NAC or vehicle group (percentage mean ± SEM, * *p* < 0.05). (**E**) Quantification of microvascular density (MVD) present on wound bed treated with NAC or vehicle (mean ± SEM, *** *p* < 0.001). (**F**) Column bar graph depicting the percentage of nuclear p-Sirt1 positive stains on keratinocytes of mice with NAC or vehicle (percentage mean ± SEM, * *p* < 0.05). (**G**) Graphic shows the percentage of ROMO1 expressing cells in keratinocytes on mice treated with NAC or vehicle (percentage mean ± SEM, ns *p* > 0.05).

**Figure 5:**
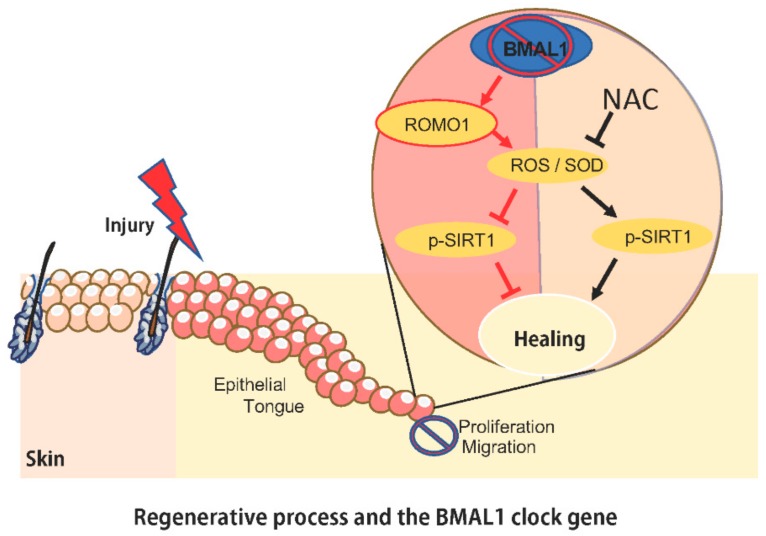
Schematic representation of BMAL1 antioxidative defense mechanism. The absence of BMAL1 leads to delayed healing due to decreased cell proliferation and migration after injury. BMAL1 ablation results in increased ROMO1, leading to the production of ROS and SOD, and decrease in p-SIRT1. ROS and SOD buildup in migrating epithelial cells reduces proliferation and migration and downregulates SIRT1. Conversely, the topical use of NAC on the skin rescued the *Bmal1*^−/−^ mice healing phenotype by blocking ROS/SOD buildup, reestablishing cellular SIRT1 activation, and ultimately resulting in cell migration, proliferation, and accelerated healing.

Our previous data indicate that the depletion of Bmal1 leads to the uncontrolled accumulation of ROS/SOD. Next, we asked if the delayed-healing phenotype observed in *Bmal1*^−/−^ mice was related to the accumulation of ROS/SOD in the skin or mediated by an alternative signaling pathway disrupted by BMAL1 deletion. Thus, we blocked the accumulation of ROS/SOD using topical NAC in *Bmal1*^−/−^ wounds. Notably, we found that the administration of NAC was sufficient to rescue the delayed-healing phenotype observed in *Bmal1*^−/−^ mice. The *Bmal1*^−/−^ mice receiving NAC had a complete wound re-epithelialization (wound closure) by day 8 after wounding (Figure 4A,B, ** *p* < 0.01). Wounds from *Bmal1*^−/−^ mice receiving the vehicle, however, remained open until day 10 (Figure 4A,B, ** *p* < 0.01). Furthermore, we found that the re-epithelialization process of NAC-treated *Bmal1*^−/−^ mice presented longer epithelial tongues compared to the mice receiving the vehicle alone (Figure 4C, ** *p* < 0.01). We also found that ROS inhibition augmented cellular proliferation (Figure 4D, *** *p* < 0.001) and remodeling of the vasculature, which indicated an advanced healing process (Figure 4E, *** *p* < 0.001). Next, we analyzed whether NAC administration affected the molecular events that follow *Bmal1* depletion. We observed that, along with the downregulation of ROS/SOD, the administration of NAC resulted in the activation of downstream signaling protein SIRT1 (Figure 4F, * *p* < 0.05). Notably, ROMO1 levels remained unaltered (Figure 4G, ns *p* > 0.05), indicating that ROMO1 is an upstream molecule from ROS/SOD and that administration of NAC has no effects over the protein levels of ROMO1 (Figure 5).

## 3. Discussion

The role of clock genes in tissue regeneration and homeostasis remains poorly understood. Our data indicate that clock gene BMAL1 is deeply involved in the process of epithelial healing, as it affects epithelial migration and proliferation. Similarly, genetic disfunction of PER, a *Bmal1* repressor gene, or the PER partner (i.e., NONO), resulted in enhanced cellular proliferation and migration [4,25,26,27]. It also became clear that BMAL1 plays a central role in controlling the levels of the reactive oxygen species during healing in a process that involves ROMO1.

ROMO1 is a transmembrane protein ubiquitously expressed in many cell types and organs, and it is responsible for promoting the production of ROS and cellular senescence [28,29]. ROMO1 is found upregulated in many tumor types, including head and neck, colorectal, and skin tumors. It is associated with poor prognosis, particularly on non-small cell lung tumors [30]. There is limited information available on the function of ROMO1 in biological processes such as tissue repair, or its involvement with clock genes. Here, we identified the involvement of ROMO1 in epidermal wound healing presenting dysfunctional BMAL1. We also showed that ROS and SOD are altered in the injured skin, particularly in animals with BMAL1 ablation.

Our data further suggest that BMAL1-induced accumulation of ROS/SOD is the prime cause of delayed tissue healing in *Bmal1*^−/−^ mice. Interestingly, NAC-induced downregulation of ROS from the epidermis did not rescue ROMO1 protein levels. Therefore, BMAL1 may exert control over ROMO1 protein levels, which precedes the ROS and SOD production. Also, it seems that there is a lack of a feedback loop mechanism linking ROS levels to ROMO1 expression. Although the balance between clock genes and the accumulation of reactive oxygen stress is starting to be elucidated, different levels of cellular stress have distinct effects over the circadian clocks. For instance, critical cellular oxidative stress levels can reset the circadian clocks, while triggering survival pathways [31]. Nevertheless, the mechanism involved in resetting the circadian clock is dependent on the BMAL1 function, which was ablated in our model.

Emerging data suggest that SIRT1 regulates clock gene expression [32]. Sirtuins have deacetylase activity (class III histone deacetylase), thereby are involved in the control of gene transcription by deacetylating modified lysines from histones and non-histones proteins, including PER2. Sirtuins play an essential role in bridging metabolism and aging [33]. Here, we observed that during healing, loss of BMAL1 leads to the dephosphorylation of SIRT1 along with the accumulation of ROS. Administration of NAC, however, resulted in the rescue of the healing phenotype along with the upregulation of phosphorylated SIRT1. The current understanding of the signaling network involving SIRT1 and clock genes suggests that BMAL1 and PER2 are dependent on SIRT1 for transcription [32]. SIRT1 binds to CLOCK and BMAL1 heterodimers, driving PER2 degradation. On the other hand, our data showed that SIRT1 levels are reduced in *Bmal1*^−/−^ mice, and that the administration of NAC rescued SIRT1 to protein levels similar to the control mice. These data suggest that reduced levels of SIRT1 observed in *Bmal1*^−/−^ mice are mediated by the accumulation of ROS and not by the depletion of *Bmal1* and potential loss of the PER2 and CLOCK-BMAL1 feedback loop control. Therefore, we have shown an alternative biologic role of the clock genes and SIRT interaction leading to the control of healing.

## 4. Materials and Methods

### 4.1. Wound Healing Assay and Experimental Mice

The experiments were carried out with *Bmal1* Knockout male mice (*Bmal1^−/−^*, B6.129-*Arntl^tm^*[1]*^Bra^*/J, Jackson Laboratory, Bar Harbor, ME, USA) and wild-type mice (control mice, colony 000664 C57BL/6J, Jackson Laboratory, Bar Harbor, ME, USA). Briefly, the mice were anesthetized and the fur from the dorsal skin was removed using an electric trimmer. The skin surface was disinfected using topical povidone-iodine solutions followed by scrubbing with gauze containing 70% ethanol. Each animal received two dorsal, bilateral, full-thickness, circular wounds using a 5 mm punch biopsy tool (extending to the panniculus carnosus). To avoid wound contraction, silicone rings were fixed around the wound with interrupted sutures and surgical glue [34]. The open wound area was monitored and measured daily using a digital caliper and digital photographs. Open wound dimensions were calculated daily as a percent of the initial wound area. NAC (3% *N*-Acetyl-l-cysteine, MilliporeSigma, St. Louis, MO, USA) was incorporated in a 2% carmellose gel base (vehicle) [35] to produce the 3% NAC gel. NAC gel was applied three times per day on the wounds. Animal procedures were approved by the Institutional Animal Care and Use Committee (IACUC; protocol PRO00009138-2019) at the University of Michigan and comply with the NIH Guidelines for the Care and Use of Laboratory Animals. Animals were housed in 12 h light/dark cycles with food and water *ad libitum* (AAALAC guidelines). The animals were observed daily by the investigators and staff.

### 4.2. Histology and Immunofluorescence

All tissues collected from the mice were fixed in aqueous buffered formalin (10% PFA), followed by dehydration using an ascending concentration of alcohol baths, and then embedded in paraffin. Embedded tissues were cut in histological sections of 4–6 µm of thickness. Representative samples of each tissue were stained with hematoxylin and eosin (H&E) using Gill’s hematoxylin (Vector, Peterborough, UK), followed by 1% eosin (MilliporeSigma, St. Louis, Mo, USA). All histological measurements were performed using H&E slides and examined under a light microscope. The process of wound re-epithelialization initiated from the wound edges and moved toward the center of the lesion. Re-epithelialization was defined as the distance traveled by the epithelium and measured in µm. Low-magnification images of the wounds were examined by two independent pathologists doing blinded analyses. Measurements took place at day 5 or indicated dates using images from a color camera (QImaging micropublisher 5.0, Surrey, BC, Canada) attached to a Nikon Eclipse 80i microscope (Nikon, Melville, NY, USA), followed by the analyses using the Nikon Elements NIS software. Immunohistochemistry was performed as previously described by us [36]. We used the primary antibodies against BrdU (Novus Biologicals, Littleton, CO, USA), α-SMA (Abcam, Cambridge, MA, USA), p-SIRT1 (Cell Signaling, Danvers, MA, USA), and ROMO1 (Thermo Fisher Scientific, Waltham, MA, USA).

### 4.3. Microvessel Density

Photomicrographs of tissue samples were used to quantify the microvessel density (MVD) of wounds using anti-CD31 antibody (1:100, Abcam, Cambridge, MA, USA) to detect microvessels. Images were captured within the wound areas using a ×20 objective to define the regions of interest (ROI). A systematic uniform random sampling (SURS) method was used to avoid observer-dependent sampling variation. Two independent examiners assessed each slide.

### 4.4. Bromodeoxyuridine (BrdU) Incorporation

Cell proliferation analysis was conducted using in vivo incorporation of 5-bromo-29-deoxyuridine (BrdU). Mice received an intraperitoneal injection of BrdU (100 mg/g) 2 h prior to sacrifice. Skin samples were fixed (10% PFA) and submitted to routine immunohistochemistry technique using anti-BrdU (Novus Biologicals, Novus Biologicals, Littleton, CO, USA) incubated at 4 °C overnight. Quantitative blinded analyses were performed by two pathologists. Results are shown as the percentage of positive cells (nuclear stain) per field.

### 4.5. Single-cell isolation from skin

The epidermis of each mouse was processed for single-cell suspension as previously described [37]. Briefly, the subcutis underlying the dorsal skin was removed using a scalpel, and both parts (dermis and epidermis) were incubated separately in Trypsin-EDTA (0.25%) for 2 h at 37 °C. Tissue was minced and passed through a 100 μm mesh filter (BD Bioscience, Franklin Lakes, NJ, USA) to achieve single-cell suspension. Cells were resuspended in cold 2% CS/PBS prior to staining.

### 4.6. ROS/SOD Assay and FACS Analysis

Single cells isolated from the skins were analyzed for reactive oxygen (ROS) and superoxide (SOD) species content using flow cytometry. Briefly, the intracellular concentration of ROS and SOD was analyzed using the ROS-ID kit (Enzo, Farmingdale, NY, USA) following the manufacturer’s instructions. Unstained cells were used as controls for endogenous fluorescence.

### 4.7. Statistical Analysis

The statistical analysis was performed using the ANOVA variance test. The Kaplan–Meier analysis was performed using the log-rank test to evaluate wound closure. T-test was used for comparisons of histological wounded size and area, epithelial tongues, and BrdU incorporation using GraphPad using GraphPrism 7 (GraphPad Software, San Diego, CA, USA). The differences were considered statistically significant for a *p*-value of *p* < 0.05. Asterisks denote statistical significance (* *p* < 0.05, ** *p* < 0.01, *** *p* < 0.001), and ns is *p* > 0.05.
